# 自噬对人肺腺癌A549细胞放疗敏感性的影响

**DOI:** 10.3779/j.issn.1009-3419.2016.12.01

**Published:** 2016-12-20

**Authors:** 丽瑶 徐, 勇 王, 琴 刘, 辉 罗, 小军 钟, 勇 李

**Affiliations:** 1 330006 南昌，南昌大学第一附属医院肿瘤科 Department of Oncology, the First Affiliated Hospital of Nanchang University, Nanchang 330006, China; 2 330006 南昌，南昌大学第一附属医院呼吸科 Department of Respirology, the First Affiliated Hospital of Nanchang University, Nanchang 330006, China

**Keywords:** 自噬, 肺肿瘤, DNA损伤修复, 放疗敏感性, Autophagy, Lung neoplasms, DNA repair, Radiosensitivity

## Abstract

**背景与目的:**

放射治疗是肺癌最重要的治疗手段之一，然而却因放疗抵抗极易导致肿瘤的复发和转移。放疗可诱导肿瘤细胞自噬发生，最新研究也报道，自噬可能与DNA损伤修复过程相关。本研究旨在探讨通过雷帕霉素上调A549细胞自噬，能否增加细胞放疗敏感性，其过程是否与DNA损伤修复过程相关。

**方法:**

以人肺腺癌A549细胞作为实验对象，实验设对照组(N)、单纯放疗组(IR)、雷帕霉素联合放疗组(R+RAPA)。采用Western blot检测γ-H2AX蛋白质、Rad51蛋白质、Ku70/80蛋白质、p62蛋白质、LC3蛋白质表达；电镜检测自噬体形成；细胞克隆形成实验检测细胞存活分数(survival fraction, SF)值。

**结果:**

与单纯放疗组相比，放疗联合雷帕霉素组自噬活性增加，且Rad51、Ku80蛋白质表达减少，细胞增殖能力下降。

**结论:**

通过雷帕霉素上调自噬可增加肺癌细胞放疗敏感性，其机制可能与抑制DNA损伤修复过程相关。

## Abstract (en)

**Background and objective:**

Radiotherapy is an important treatment for lung cancer. The poor prognosis of lung cancer is largely caused by the high recurrence rate and metastasis of the tumor. Autophagy, which can be induced by radiotherapy, might be associated with DNA repair. The aim of this study is to investigate whether activating autophagy using rapamycin can enhance the radiosensitivity of lung cancer cells and clarify the association of autophagy with DNA repair.

**Methods:**

The human adenocarcinoma A549 cell line was selected as the experimental subject. The specimens were divided into three groups: control (N), radiation (R), and Rapamycin and radiation (R+RAPA). The protein levels of γ-H2AX, Rad51, Ku70/Ku80, p62, and LC3 were determined by Western blot. Autophagosome was observed under a transmission electron microscope, and SF was determined by colony formation assay.

**Results:**

Compared with group R, the activity of autophagy and the protein expression levels of Rad51 and Ku70/80 were remarkably increased in group R+RAPA.

**Conclusions:**

The radiosensitivity of lung cancer can be promoted by activating autophagy via treatment with Rapamycin, and the process may be associated with DNA repair.

肺癌是严重危害人类健康的疾病，据美国癌症学会2011年公布的统计数据表明，肺癌的发病率(160万/年)及死亡率(140万/年)均居全球癌症首位^[1]^。放疗是肺癌最重要的治疗手段之一，其机制主要是通过放疗射线引起DNA损伤^[2]^。由于肿瘤细胞具有较强的DNA损伤修复能力，严重限制了肺癌放射治疗的效果。DNA损伤主要通过同源重组修复(homologous recombination, HR)和非同源末端连接(nonhomologous end joining, NHEJ)两种方式进行修复。其中Rad51是HR修复的关键分子^[3]^，而Ku70/80是启动和调节NHEJ的关键因子^[4]^，其中任一组分的表达改变或功能缺失，都可能改变DNA损伤修复效率。

最近，有研究^[5-7]^报道自噬可影响DNA损伤修复过程。自噬是真核细胞中常见的自我消化现象，在大部分肿瘤细胞中，自噬处于较低的基础水平，一旦处于应激状态下(如缺氧、能量不足、放疗射线)，自噬水平可能急剧上调^[8, 9]^。为了探究自噬是否影响肺癌A549细胞的放疗敏感性，其分子机制是否可能与DNA损伤修复过程相关，本研究采用雷帕霉素(rapamycin, RAPA)上调A549细胞自噬水平，观察自噬活化时，放疗射线对A549细胞存活和增殖活性的影响，以及DNA损伤修复水平的改变，明确是否可以通过雷帕霉素调控自噬进而影响肺癌A549细胞的放疗敏感性，并初步分析DNA损伤修复过程是否与其相关，以探讨通过调控自噬增强局部放射治疗效果的可能性，为提高肺癌患者的治疗效果及远期生存率提供理论基础。

## 1 材料与方法

### 1.1 试剂及仪器

RPMI-1640培养基，胎牛血清(Hyclone)，蛋白质提取试剂盒(Pierce公司)，PCR仪(ABI)，LC3抗体(Sigma)，p62抗体(BD bioscience)，γ-H2AX抗体(Abcam公司)，Rad51抗体、Ku70/80抗体(CST)，Trizol(Transgene)，逆转录试剂盒、2×SYBR试剂盒(Takara)，蛋白质电泳仪、电转仪(Bio-Rad)，直线加速器(西门子)。

### 1.2 细胞培养、照射方式及实验分组

#### 1.2.1 细胞培养

人肺腺癌A549细胞(购于中科院上海细胞库)，用含10%胎牛血清的RPMI-1640培养基于37 ℃，常规传代培养。

#### 1.2.2 照射方式

照射条件：室温条件下采用6 MV X线照射细胞，培养皿上方垫1.5 cm有机玻璃板。源皮距为100 cm，照射剂量为4 Gy(剂量率为200 cGy/min)，每个实验组设置3个平行样本。

#### 1.2.3 实验分组

本研究设①对照组；②单纯放疗组；③放疗+RAPA组。取生长状态良好的A549细胞经0.25%胰酶消化，待细胞生长密度约70%-80%时，对照组不经任何处理，单纯放疗组加入DMSO，放疗+RAPA组加入100 nM RAPA后继续培养24 h，单纯放疗组及放疗+RAPA组行4 Gy放疗射线照射。

### 1.3 电镜下观察自噬体的变化

采用不含EDTA的胰酶消化细胞，PBS洗3次，加入新配的4%多聚甲醛，4 ℃过夜。经饿酸进一步固定、系列丙酮脱水、浸透、包埋、超薄切片、染色后于透射电镜下观察自噬体形成情况。

### 1.4 Western blot检测蛋白质表达

冷PBS洗2次，加入细胞裂解液于4 ℃裂解20 min，13, 000 g离心20 min，收集上清，提取细胞总蛋白质，BCA法测蛋白质浓度。取20 µg样品煮沸变性后进行Tricine-SDS-PAGE电泳，转膜，5%脱脂奶粉37 ℃封闭2 h，一抗4 ℃孵育过夜，洗膜，二抗37 ℃孵育1 h，ECL法显影，分析条带灰度值，以目的条带与β-actin调对灰度比值来表示蛋白质相对表达量^[10, 11]^。

### 1.5 细胞克隆形成实验检测放疗对A549细胞增殖的影响

放疗后经胰酶消化，收集细胞后，经细胞计数，将细胞接种于含5 mL预温37 ℃培养基的60 mm培养皿中，以十字方向轻轻晃动培养皿，使细胞分散均匀。常规培养2周后，终止培养，结晶紫染色。根据以下公式计算克隆形成率及细胞存活分数：克隆形成率(plating efficiency, PE)=(每组平均集落数/每皿接种的细胞数)×100%，细胞存活分数(survival fraction, SF)=(实验组集落形成率/对照组集落形成率)×100%。

### 1.6 统计学方法

采用SPSS 17.0统计软件进行分析，各组的数据以均数±标准差表示，两组间均数比较采用*t*检验，多样本均数比较采用单因素方差分析。以*P* < 0.05为差异有统计学意义。

## 2 结果

### 2.1 雷帕霉素调控自噬影响A549细胞的放疗敏感性

电镜检测发现，与单纯放疗组相比，雷帕霉素联合放疗组自噬小体形成增加(图 1A)。此外，我们还通过Western blot检测自噬底物蛋白质p62及自噬标志性蛋白质LC3表达。经图像半定量分析灰度值，结果发现，与对照组相比，放疗组LC3Ⅱ/LC3Ⅰ表达增加为(4.1±0.29)倍，p62表达减少为(0.83±0.12)，放疗联合雷帕霉素组LC3Ⅱ表达增加为(5.8±0.32)，p62表达减少为(0.42±0.06)，(图 1B，图 1C)(*P* < 0.05)。通过克隆形成实验，我们还发现，与对照组相比，单纯放疗组SF减少为(0.61±0.12)，放疗联合雷帕霉素组SF减少为(0.37±0.04)(图 1D)(*P* < 0.05)。

**1:**
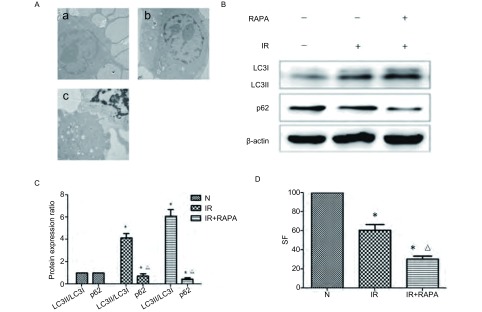
Rapamycin通过诱导自噬增加放疗敏感性。A：A549细胞给予Rapamycin(100 nM)预处理24 h，放疗(4 Gy)后4 h收集细胞，采用电镜(10, 000×)检测自噬体。A：a：正常组；b：IR组；c：IR+RAPA组。B：采用Western blot检测Rapamycin(100 nM)预处理24 h，放疗(4 Gy)后4 h LC3蛋白质、p62蛋白质表达变化。C：QuanlityOne软件进行半定量分析，LC3蛋白质、p62蛋白质表达倍数变化。D：细胞克隆形成实验检测Rapamycin(100 nM)预处理24 h，放疗后A549细胞增殖情况。与N组相比，^*^P < 0.05，与IR组相比，^△^*P* < 0.05。 Rapamycin sensitized radiation by induced autophagy. A: A549 cells were incubated with 100 nmol/L rapamycin for 24 h before exposure to 4 Gy of irradiation, cells were extracted at 4 h after irradiation, autophagosomes were observed under transmission electron microscope (10, 000×). A: a: Control group; b: IR group; c: IR+RAPA group. B: A549 cells were incubated with 100 nmol/L rapamycin for 24 h before exposure to 4 Gy of irradiation, proteins were extracted at 4 h after irradiation, LC3Ⅱ/Ⅰ and p62 expression were detected by Western blot analysis. C: Semi-quantitated of LC3Ⅱ/Ⅰ and p62 expression using QuanlityOne software. D: The survival fraction of rapamycin-treated A549 cells for 24 h after exposure IR were analyzed by clonogenic assay. ^*^P < 0.05 *vs* Control group, ^△^*P* < 0.05 *vs* IR group.

### 2.2 雷帕霉素通过调控自噬影响A549细胞放疗敏感性的机制

γ-H2AX为DNA损伤标志性分子，Western blot检测发现，放疗后1 h，与对照组相比，单纯放疗组γ-H2AX蛋白质表达增加为(9.6±0.21)，放疗联合雷帕霉素组γ-H2AX蛋白质表达增加为(10.8±0.17)(*P* < 0.05)，单纯放疗组与放疗联合雷帕霉素组之间无统计学差异；放疗后24 h单纯放疗组γ-H2AX蛋白质表达下调为(1.8±0.04)，放疗联合雷帕霉素组γ-H2AX蛋白质表达下调为(4.3±0.07)(*P* < 0.05)(图 2A，图 2B)。

**2:**
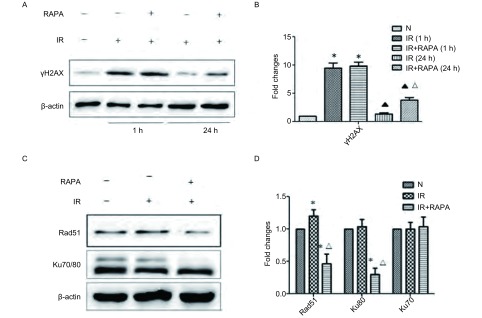
Rapamycin通过自噬抑制DNA损伤修复，增加放疗敏感性。A：A549细胞给予Rapamycin(100 nM)预处理24 h，放疗(4 Gy)后1 h、24 h收集细胞，采用Western blot检测*γ*-H2AX蛋白质表达变化。B：QuanlityOne软件进行半定量分析，*γ*-H2AX蛋白质表达倍数变化。与N组相比，^*^P < 0.05。与1 h相比，^▲^*P* < 0.05；与IR组相比，^△^*P* < 0.05。C：处理同前，于放疗后4 h收集细胞，采用Western blot检测Rad51蛋白质、Ku70/80蛋白质表达。D：QuanlityOne软件进行半定量分析，Rad51、Ku70蛋白质表达变化。与N组相比，^*^P < 0.05；与IR组相比，^△^*P* < 0.05。 Rapamycin sensitized radiation by autophagy inhibited the process of DNA damage repair. A: A549 cells were incubated with 100 nmol/L rapamycin for 24 h before exposure to 4 Gy of irradiation, proteins were extracted at 4 h after irradiation, *γ*-H2AX expression were detected by western blot analysis. B: Semi-quantitated of *γ*-H2AX expression using QuanlityOne software. ^*^P < 0.05 *vs* Control group, ^▲^*P* < 0.05 *vs* 1 h, ^△^*P* < 0.05 *vs* IR group. C: Treated as before, cells were extracted at 4 h after irradiation, LC3Ⅱ/Ⅰ and p62 expression were detected by western blot analysis. D: Semi-quantitated of Rad51 and Ku70 expression using QuanlityOne software. ^*^P < 0.05 *vs* Control group, ^△^*P* < 0.05 *vs* IR group.

Western blot检测发现，与对照组相比，放疗组Rad51蛋白质表达增加为(1.2±0.04)(*P* < 0.05)，Ku70蛋白质、Ku80蛋白质表达无明显变化，放疗联合雷帕霉素组Rad51蛋白质表达减少为(0.4±0.03)，Ku80蛋白质表达减少为(0.3±0.08)(*P* < 0.05)，Ku70无明显变化(图 2C，图 2D)。

## 3 讨论

DNA损伤修复系统在维护基因组的完整与稳定中起着至关重要的作用，肿瘤细胞的DNA损伤修复是最终导致放射性抵抗的罪魁祸首，极大地影响了肺癌放射治疗的效果。有研究^[12]^表明，干预DNA修复路径将削弱肿瘤细胞基因组的稳定，造成肿瘤细胞基因片段的缺失、遗传突变、甚至细胞的死亡。但是，目前有关于自噬与放疗敏感性的研究大多关注于通过调控自噬上游通路影响放疗敏感性，而自噬对肺癌放疗后DNA损伤修复的影响及其相关分子机制的研究甚少。因此，了解自噬是通过何种途径促进放疗后细胞死亡，可为放射治疗提供新的切入点和思路，对逆转肺癌的放疗抵抗治疗具有重要意义^[13]^。

本研究选取人肺腺癌A549细胞作为研究对象，我们前期已通过MTT实验观察了不同浓度雷帕霉素在不同作用时间对A549细胞增殖的影响，结合相关文献[14-16]报道，最终选用100 nmol/L剂量浓度的雷帕霉素作用A549细胞24 h，仅有10% A549细胞生长受抑制，最大限度减少了雷帕霉素药物本身对A549细胞的杀伤作用，以利于本项目观察雷帕霉素对A549细胞的放疗增敏作用。p62为自噬底物，自噬发生时，p62表达减少；而LC3存在两种形式，LC3Ⅰ和LC3Ⅱ，前体LC3合成后经加工形成LC3Ⅰ，后者与自噬体膜上的磷脂酰乙醇胺结合形成LC3Ⅱ，当自噬形成时，LC3Ⅰ减少而LC3Ⅱ增加。透射电镜是检测自噬的金标准。我们通过Western blot及电镜发现放疗射线可诱导A549细胞自噬发生，且雷帕霉素可进一步上调放疗所诱导的自噬。克隆形成实验证实雷帕霉素联合放疗后SF值较单纯放疗组下降。以上结果表明通过雷帕霉素上调人肺腺癌A549细胞自噬水平，可促进放疗后A549细胞死亡，增加其放疗敏感性，减少其放疗抵抗，具有放疗增敏作用。

自噬联合放疗有望成为肺癌治疗的新策略，放射治疗的敏感性主要与放疗后细胞DNA损伤的修复程度相关，目前，少量研究报道，自噬可影响细胞DNA损伤修复过程，Robert等^[6]^在酵母中发现，自噬通过降解乙酰化的DSBs修复酶ctlp、Exo1，阻断HR修复途径。然而，自噬是否可通过影响DNA损伤修复过程增加肺癌的放疗敏感性，目前尚不明确。

在本研究中，我们发现，放疗后1 h，与对照组相比，放疗组及放疗联合雷帕霉素组γ-H2AX蛋白质(DNA损伤标志分子)表达上调，但两组之间无明显差异，放疗后24 h，单纯放疗组及放疗联合雷帕霉素组γ-H2AX蛋白质(DNA损伤标志分子)表达下调，且单纯放疗组减少更明显。放疗射线可诱导A549细胞DNA损伤，但由于A549细胞具有较强的DNA损伤修复能力，限制了放疗射线对A549细胞的杀伤作用，同时给予雷帕霉素处理，虽并未明显增加放疗后A549细胞DNA损伤，然γ-H2AX持续存在，DSBs未得到正确有效的修复，损伤DNA的数量增加，从而增加肿瘤放射治疗的有效性。

放疗后DNA损伤修复主要依赖以下两种途径，HR修复和NHEJ修复^[17]^，其中HR修复是细胞发生DNA损伤后进行精确修复的主要机制，其具体机制十分复杂，涉及大量蛋白质，最重要的是Rad51；而NHEJ修复则是细胞发生DNA损伤后进行修复的最为普遍机制，DNA-PK是参与NHEJ途径要的分子之一，DNA-PK由催化亚基DNA-PKCs和两个调节亚基Ku70、Ku80组成，其中Ku70和Ku80作为辅助因子，在DNA损伤修复过程中发挥着重要作用，其功能可能较DNA-PKCs更为广泛^[18]^。大量研究证实，哺乳动物细胞内缺乏Rad51、Ku70、Ku80时，DSBs修复能力下降，细胞对放疗的敏感性增加。Zhou等^[19]^的研究证实采用siRNA敲除E3泛素连接酶RNF8，可通过减少Rad51表达，进而增加肺腺癌A549细胞放疗敏感性。Belenkov等^[20]^采用反义核苷酸技术减少Ku蛋白质表达，减少DNA损伤修复，增加脑胶质瘤细胞放疗敏感性。Chen等^[21]^的研究还发现，雷帕霉素可减少Rad51蛋白质至DNA损伤部位的聚集，从而起到放疗增敏的作用。我们的研究发现，采用雷帕霉素上调自噬增加放疗敏感性的同时，放疗后Rad51蛋白质、Ku80蛋白质表达较单纯放疗组下降，以上结果表明，通过雷帕霉素上调肺腺癌A549细胞自噬，可起到放疗增敏的效果，鉴于雷帕霉素可影响Rad51及Ku80蛋白质水平，我们推测，通过雷帕霉素上调自噬可促进放疗射线对肺腺癌A549细胞的杀伤作用，增加放疗敏感性，以上过程可能与DNA损伤修复的同源重组及非同源末端连接相关，其具体机制有待进一步研究。

综上所述，在体外细胞实验中，雷帕霉素可通过上调A549细胞自噬水平，促进DSBs修复相关蛋白质降解，从而增加放疗敏感性。提示自噬激活剂或可被用于肺癌的辅助治疗，目前我们仅仅进行了体外研究，初步分析了自噬影响A549细胞放疗敏感性的可能机制，但其深入的机制及作用尚有待更多的体内外研究进行验证。
